# Application of a rehabilitation program for executive functions in a sample of Egyptian children with learning disorder

**DOI:** 10.1186/s43163-023-00391-6

**Published:** 2023-02-03

**Authors:** Engy Samy Elhakeem, Soha Abd Elatif Ahmed Ibrahim, Riham Mohamed El-Maghraby, Ahmed Abd El Aal Fouad

**Affiliations:** 1 Unit of Phoniatrics, Department of Otorhinolaryngology, Faculty of Medicine, Alexandria University, Alexandria, Egypt; 2 Department of Neuropsychiatry, Faculty of Medicine, Alexandria University, Alexandria, Egypt; 3 Department of Otorhinolaryngology, Faculty of Medicine, Alexandria University, Alexandria, Egypt

**Keywords:** Executive functions, Learning disorder, Children, Dyslexia, ADHD

## Abstract

**Background:**

In recent years, significant progress has been made on ways to improve executive function (EF) skills for school readiness involving direct EF training and classroom educational programs. Due to the absence of a well-structured Arabic program for EF training in children, the rationale of this study is to implement a comprehensive, evidence-based intervention program to help Egyptian children with learning disorders to overcome their EF impairment. It uses the multimodality approach to help meet the needs of students with a variety of learning styles. The aim of this study is to adapt the combined form of the “Executive Functions Training-Elementary” and the “Promoting Executive Function In The Classroom” programs and its application to test its effectiveness in the rehabilitation of Egyptian learning-disordered children.

**Results:**

The results showed significant improvement in the Arabic version of Barkley Deficits in Executive Functioning Scale, Children and Adolescents long-form (BDEFS-CA) scores for executive functions (EF) evaluation (executive function summery score from 228.63 to 213.77 with *p*-value < 0.001), and it also showed significant improvement in the Arabic dyslexia assessment test (ADAT) scores (from 1.89 to 1.33 with *p*-value < 0.001) for dyslexia evaluation.

**Conclusion:**

The study concluded that designing an Arabic rehabilitation program specific for executive function difficulties was effective for improving both EF deficits and dyslexia, but there is a need for further studies comparing this program to other methods of traditional interventions.

**Trial registration:**

The study was retrospectively registered at www.clinicaltrials.gov NCT05476133, approved on 26 July 2022. Register name is the following: application of a rehabilitation program for executive functions in a sample of Egyptian children with learning disorder.

## Background

Executive functions (EF) are considered a group of cognitive abilities that enable individuals to perform targeted, purposeful behaviors and to adapt to novel situations, with conscious control. They are used as an umbrella term that includes a diverse group of hypothesized cognitive processes that have interrelated functions [1].

Various conceptual models have been proposed to explain EF processes, although none has been universally adopted [2, 3]. It has been found recently that working memory, inhibition, and cognitive flexibility are the core EF skills from which higher-order EF skills (e.g., planning and problem-solving) are built [4].

Students who suffer from specific learning disorder (SLD) can also have an elevated risk of executive function difficulties (EdF) [5, 6]. During the elementary period, children with EdF may demonstrate difficulties in reading, written expression, and mathematical skills, and later on, these difficulties manifest more evidently in the inability to plan, organize, complete projects, and perform well in exams [5]. For instance, it was recently proved that dyslexia is associated with global executive function difficulties, with more errors in inhibitory control (commission errors), more perseverative errors, higher response variability, and lower consistency of responses over time [1, 7].

Indeed, in these cases, developing EF skills requires continued efforts in the home and school environment by parents and teachers [6]. In recent years, significant progress has been made on ways to improve EF skills for school readiness involving direct EF training and classroom educational programs [8, 9].

Due to the absence of a well-structured Arabic program for EF training in children, the rationale of this study is to incorporate ideas from the “Executive Functions Training-Elementary” [10] and the “Promoting Executive Function In The Classroom” programs [11] to help Egyptian children with learning disorder to overcome their EF impairment. It uses the multimodality approach to help meet the needs of students with a variety of learning styles.

## Methods

### Aim of the work

It is formulation of a rehabilitation program for adaptation of therapy activities for improving EF deficits and overall academic performance and application of this program in cases with EdF to test its efficiency in improving EF deficits and academic skills in Egyptian Arabic-speaking children.

### Patients

This study was carried out on 40 children with specific learning disorder (SLD) and executive function deficits of both sexes and aged from 6 to 11 years attending the Unit of Phoniatrics, in the outpatient clinic of Alexandria Main University Hospital. Children with brain damage, mentally retarded, a history of clinical and subclinical fits, hearing impairment or visual impairment, and psychiatric problems were excluded from the study.

The ethics committee of the Faculty of Medicine, Alexandria University (IRB No: 00012098, FWA No: 00018699), approved the study (0,106,137). The study was retrospectively registered at www.clinicaltrials.gov NCT05476133.

An informed consent was orally taken from parents and/or legally caring surrogates in addition to the child’s assent of all children participating in the study.

### Methodology

The following intervention study proceeded in the following order:

1. *Formulation* of the remediation program by using a combined form of the “Executive Functions Training-Elementary”[10] and the “Promoting Executive Function In The Classroom” [11] rehabilitation programs which were translated and modified to be used for Egyptian children with learning disorders.
2. *Initial assessment*, including detailed history taking and thoughtful general and neurological examination.
3. *Clinical diagnostic procedures*, including psychometric evaluation as follows: Stanford-Binet scale 4th edition to assess intelligence quotient and mental age [12]; children’s attention and adjustment survey (CAAS) test for cases complaining of attention-deficit/hyperactivity disorder (ADHD) symptoms [13]; Arabic dyslexia assessment test (ADAT) for the diagnosis of dyslexia [14]; auditory assessment for exclusion of audiological problems; ophthalmological assessment for exclusion of visual problems; and evaluation of EFs by the Arabic version of Barkley Deficits in Executive Functioning Scale, Children and Adolescents long-form (BDEFS-CA) [15].
4. *Intervention*: All children received the modified combined form of the “Executive Functions Training-Elementary” [10] and the “Promoting Executive Function In The Classroom” rehabilitation programs [11] for 2 sessions per week (45 min to 1 h per session) for a period between 3 and 6 months depending on the response of the child.

Modifications to the program were done for adaptation with the Arabic language and the Egyptian dialect, including the following:

- Acronyms, initializations, and silly sentences: Examples and exercises 2 (point 4), 3 (points 2 and 3), and 5 (points 2 and 3)
- Creating homework plans: Exercises 1 (point 3), 2 (point 7), 3 (point 6), 4 (points 3 and 4), and 5 (point 8)
- Studying for tests in advance: Exercise 5
- Brainstorming: Examples for webs and list — exercise 1 (point 3)
- Reading with a purpose: Exercise 2 (point 1)
- Unscrambling and reordering sentences: All sentences were modified to conform to Arabic language syntax.
- Homographs and syllable stress: All words, sentences, and paragraphs were modified to conform to Arabic language syntax.
- Editing written work: All sentences and paragraphs were modified to conform to Arabic language syntax.

All names of people and places were changed to Arabic names to suit the Egyptian children.

5) *Reevaluation*: Was done after 3–6 months of therapy to test the effectiveness of the program in the rehabilitation of Egyptian learning disordered children.

### Data management and statistical analysis

Data were collected and entered the computer using SPSS (Statistical Package for Social Science) program for statistical analysis (version 21). Data were entered as numerical or categorical, as appropriate. Kolmogorov–Smirnov test of normality revealed no significance in the distribution of the variables, so the parametric statistics were adopted. Data were described using minimum, maximum, mean, standard deviation, and 95% CI of the mean. Categorical variables were described using frequency and percentage. Comparisons were carried out between two studied dependent normally distributed variables using paired *t*-test. Comparisons were carried out between more than two independent normally distributed subgroups using the one-way ANalysis Of VAriance (ANOVA) test. When the F-ratio of ANOVA was significant, Levene’s test of homogeneity of variances was done, and if significant, Brown-Forsythe robust test was adopted. Post hoc multiple comparisons were done using Games-Howell. Pearson’s correlation was done. The confidence interval was set to 95%, and the margin of error accepted was set to 5%. So, the *p*-value was considered significant if < 0.05.

## Results

The mean age of the studied children was 8.99 ± 1.27 years, and 74.3% were males. Table 1 illustrates the distribution of the study sample according to detailed demographic data and history.

**Table 1:** Distribution of the studied cases according to demographic data and history

	**No**			**%**
**Sex**				
Male	26			74.29
Female	9			25.71
**Age (years)**				
Min.-max	6.08–10.92			
Mean ± SD	8.99 ± 1.27			
**Consanguinity**				
Negative		32	91.43	
Positive		3	8.57	
**Handedness**				
Right-handed		32	91.43	
Left-handed		3	8.57	
**Family history**				
No		26	74.29	
Yes		9	25.71	
**If yes, who**				
Father		2	22.22	
Sibling		2	22.22	
Uncle		1	11.11	
Cousin		4	44.44	
**Grade**				
1st		2	5.71	
2nd		12	34.29	
3rd		5	14.29	
4th		11	31.43	
5th		5	14.29	
**School**				
Governmental		25	71.43	
Experimental		6	17.14	
Private		4	11.43	
**Education**				
Normal		29	82.86	
Integrated		6	17.14	
**History of DLD**				
Negative		18	51.43	
Positive		17	48.57	
**Age of first word (months)**				
Min–max		9.00–36.00		
Mean ± std. deviation		17.57 ± 7.83		
**Auditory assessment**				
Not impaired		34	97.14	
Impaired		1	2.86	
**Ophthalmological assessment**				
Not impaired		28	80.00	
Impaired		7	20.00	

Table 2 shows that in all the Stanford-Binet psychometric test (4th edition), domains, the majority of cases were in the average area.

**Table 2:** Distribution of the studied cases according to the intelligence quotient (IQ) (*n* = 35)

		**Mild MR (55–69)**	**Borderline (70–79)**	**Below average (80–89)**	**Average (90–110)**	**Above average (111–119)**
**Stanford-Binet VIQ**						
Min–max Mean ± std. deviation	74.00–104.00 90.14 ± 7.37	-	2 (5.71%)	10 (28.57%)	23 (65.71%)	-
**Stanford-Binet AIQ**						
Min–max Mean ± std. deviation	76–118 94 ± 9	-	1 (2.86%)	8 (22.86%)	24 (68.75%)	2 (5.71%)
**Stanford-Binet STM**						
Min–max Mean ± std. deviation	65–100 85 ± 9	3 (8.57%)	5 (14.29%)	13 (37.14%)	14 (40.00%)	-
**Stanford-Binet GIQ**						
Min–max Mean ± std. deviation		-	3 (8.57%)	12 (34.29%)	20 (57.14%)	-

*VIQ* verbal intelligence quotient, *AIQ* abstract intelligence quotient, *STM* short-term memory, *GIQ* general intelligence quotient

Table 3 shows that in all the CAAS psychometric test domains, most cases were affected.

**Table 3:** Distribution of the studied cases according to the children’s attention and adjustment survey (CAAS) test results (*n* = 35)

		**Not affected**	**Affected**
**CAAS N**			
Min–max Mean ± std. deviation	35–100 72 ± 18	11 (31.43%)	24 (68.57%)
**CAAS I**			
Min–max	27–100	8	27
Mean ± std. deviation	71 ± 19	(22.86%)	(77.14%)
**CAAS ADD**			
Min–max	29–99	9	26
Mean ± std. deviation	71 ± 17	(25.71%)	(74.29%)
**CAAS H**			
Min–max Mean ± std. deviation	29–99 66 ± 16	13 (37.14%)	22 (62.86%)
**CAAS ADHD**			
Min–max Mean ± std. deviation	16–100 66 ± 21	10 (28.57%)	25 (71.43%)
**CAAS CD**			
Min–max Mean ± std. deviation	22–99 63 ± 18	16 (45.71%)	19 (54.29%)

*CAAS N*, CAAS inattention; *CAAS I*, CAAS impulsivity; *CAAS ADD*, CAAS attention-deficit disorder; *CAAS H*, CAAS hyperactivity; *CAAS ADHD*, CAAS attention-deficit hyperactivity disorder; *CAAS CD*, CAAS conduct disorder

Distribution of the sample before and after intervention according to the presence or absence of a specific learning disorder (SLD) in the form of dyslexia was that before intervention, all the study cases were affected, while after intervention, 28.57% of the cases were completely improved. There was a statistically significant decrease in the test score after intervention with a *p*-value of < 0.001 as the mean score after intervention was 1.33 ± 0.65, while before intervention, it was 1.89 ± 0.59 (Table 4).

**Table 4:** Distribution of the studied cases according to the ADAT before and after intervention (*n* = 35)

	**Group**	**Not affected**	**Affected**	**Test of significance** ***p*-value**
**ADAT pretest**				*p* = < 0.001*
Min–max Mean ± std. deviation	1.00–2.80 1.89 ± 0.59	0 (0.00%)	35 (100.00%)	
**ADAT posttest**				
Min–max Mean ± std. deviation	0.10–2.60 1.33 ± 0.65	10 (28.57%)	25 (71.43%)	

*ADAT*, Arabic dyslexia assessment test

*p*, *p*-value for comparing between **pre** and **post**. *Statistically significant at *p* < 0.05

^**^Highly significant at *p* < 0.001

Table 5 shows that the distribution of the study sample according to BDEFS-CA results was illustrated before and after intervention as not affected, mild, moderate, or severe. Overall, the executive function summary score (EFSS) component of the BDEFS-CA test showed that there was a statistically significant decrease in the test score after intervention with a *p*-value of < 0.001 as the mean score after intervention was 213.77 ± 22.37, while before intervention, it was 228.63 ± 23.52.

**Table 5:** Distribution of the studied cases according to the BDEFS-CA before and after intervention (*n* = 35)

	**Group**	**Not affected**	**Mild**	**Moderate**	**Severe**	**Test of significance** ***p*-value**
**BDEFS-CA pretest SMT**						*p* = < 0.001*
Min–max Mean ± std. deviation	36.00–54.00 45.09 ± 4.83	1 (2.86%)	15 (42.9%)	13 (37.14%)	6 (17.14%)	
**BDEFS-CA posttest SMT**						
Min–max Mean ± std. deviation	33.00–53.00 41.40 ± 4.78	12 (34.29%)	17 (48.57%)	4 (11.43%)	2 (5.71%)	
**BDEFS-CA pretest SO**						*p* = < 0.001*
Min–max Mean ± std. deviation	28.00–53.00 39.00 ± 6.15	1 (2.86%)	16 (45.71%)	13 (37.14%)	5 (14.29%)	
**BDEFS-CA posttest SO**						
Min–max Mean ± std. deviation	30.00–55.00 36.74 ± 5.33	1 (2.86%)	25 (71.43%)	7 (20.00%)	2 (5.71%)	
**BDEFS-CA pretest SR**						*p* = < 0.001*
Min–max Mean ± std. deviation	34.00–50.00 42.91 ± 3.94	1 (2.86%)	17 (48.57%)	14 (40.00%)	3 (8.57%)	
**BDEFS-CA posttest SR**						
Min–max Mean ± std. deviation	32.00–49.00 39.89 ± 4.28	10 (28.57%)	17 (48.57%)	8 (22.86%)	-	
**BDEFS-CA pretest SM**						*p* = < 0.001*
Min–max Mean ± std. deviation	40.00–55.00 47.46 ± 4.55	2 (5.71%)	14 (40.00%)	15 (42.86%)	4 (11.43%)	
**BDEFS-CA posttest SM**						
Min–max Mean ± std. deviation	36.00–53.00 44.11 ± 4.69	7 (20.00%)	20 (57.14%)	7 (20.00%)	1 (2.86%)	
**BDEFS-CA pretest SRE**						*p* = < 0.001*
Min–max Mean ± std. deviation	39.00–63.00 54.46 ± 6.0	3 (8.57%)	12 (34.29%)	16 (45.71%)	4 (11.43%)	
**BDEFS-CA posttest SRE**						
Min–max Mean ± std. deviation	37.00–63.00 51.63 ± 6.18	7 (20.00%)	15 (42.86%)	12 (34.29%)	1 (2.86%)	
**BDEFS-CA pretest EFSS**						*p* = < 0.001*
Min–max Mean ± std. deviation	191.00–270.00 228.63 ± 23.52	0 (0.00%)	14 (40.00%)	14 (40.00%)	7 (20.00%)	
**BDEFS-CA posttest EFSS**						
Min–max Mean ± std. deviation	181.00–271.00 213.77 ± 22.37	5 (14.29%)	17 (48.57%)	10 (28.57%)	3 (8.57%)	

*BDEFS-CA*, Barkley Deficits in Executive Functioning Scale, Children and Adolescents long form. *SMT*, self-management to time; *SO*, self-organize; *SR*, self-restraint; *SM*, self-motivate; *SRE*, self-regulation of emotion; *EFSS*, executive function summary score

*p p*-value for comparing between **pre** and **post**. *Statistically significant at *p* < 0.05

^**^Highly significant at *p* < 0.001

**Fig. 1:**
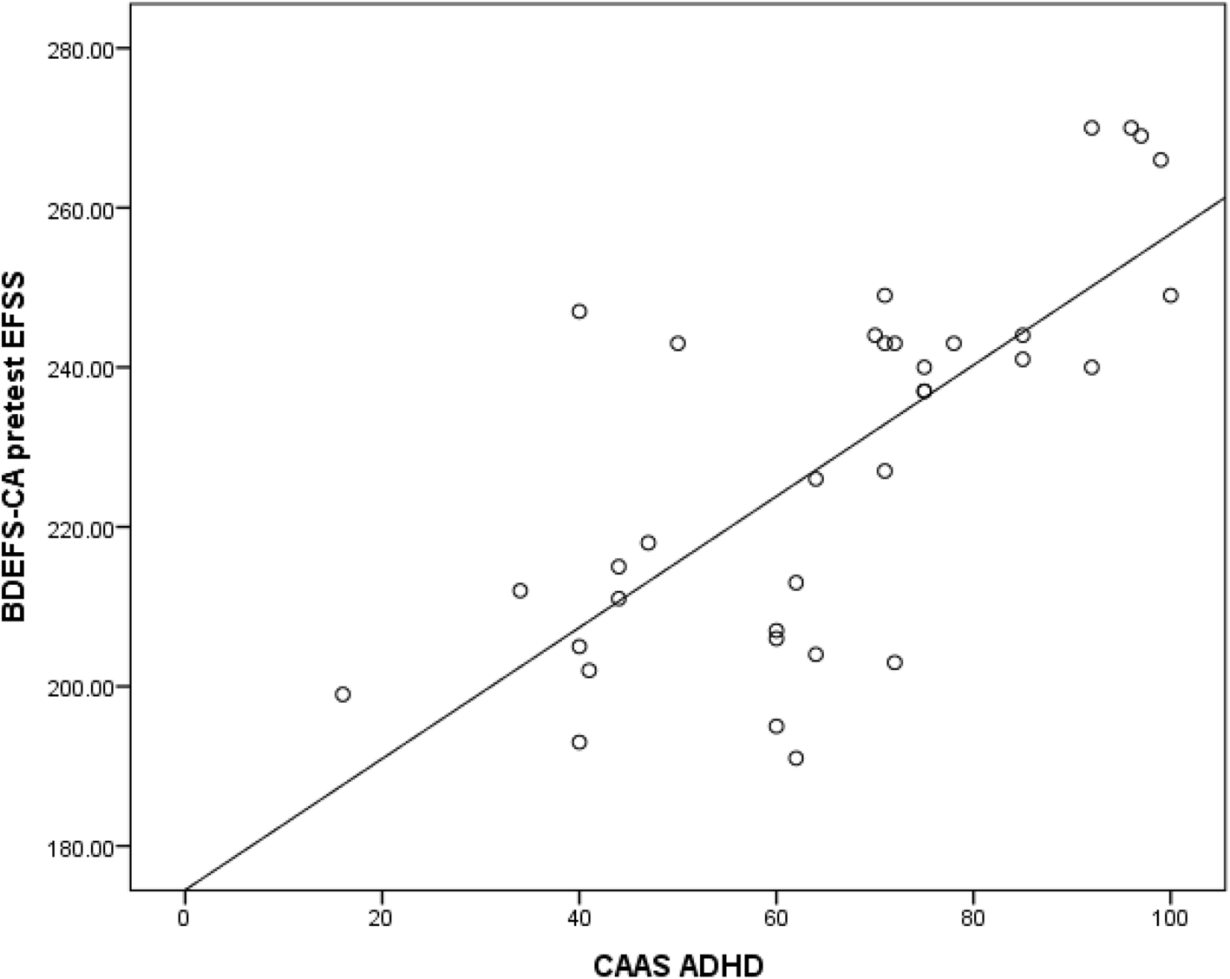
Correlation between BDEFS-CA pretest EFSS and CAAS ADHD

In this study, a positive correlation was found between BDEFS-CA pretest overall summary score (EFSS) and CAAS ADHD (Fig. 1).

Correlations between ADAT percentage change (before and after intervention) and BDEFS-CA different categories percentage change (before and after intervention) were all significantly positive (Table 6).

**Table 6:** Correlation between ADAT percentage change and BDEFS-CA percentage change

	**ADAT percentage change**	
	***r***_**s**_	***p***
**BDEFS-CA SMT percentage change**	− 0.539	0.001*
**BDEFS-CA SO percentage change**	− 0.499	0.002*
**BDEFS-CA SR percentage change**	− 0.395	0.019*
**BDEFS-CA SM percentage change**	− 0.142	0.417 NS
**BDEFS-CA SRE percentage change**	− 0.447	0.007*
**BDEFS-CA EFSS percentage change**	− 0.585	< 0.001*

*r*, Pearson coefficient. *Statistically significant at *p* < 0.05. **Statistically highly significant at *p* < 0.001

## Discussion

There is a general agreement among most researchers that a combination of diverse skills helps students in a successful transition to formal schooling such as language, early literacy and math skills, EF, social-emotional skills, and visual-motor integration [16]. Accordingly, this study aimed to design a rehabilitation program in Arabic language to test its efficacy in improving deficits in EFs and overall academic performance.

It was found that the majority of the samples (74.29%) were males, and this may be explained by the higher incidence of dyslexia among males as compared to the study done by Akyurek et al. 2019 [7], which reported a male percentage of 65.2%.

The mean age was 8.99 years and standard deviation 1.27 years which may be attributed to the increased demands of EF in the middle childhood period; furthermore, this age group is the most frequently referred for the rehabilitation of dyslexia, and this is consistent with the findings of Cantin et al. (2016) [17], which reported a mean age of 9 years and standard deviation of 1.16 years. The literature review did not present relevant information about the effects of sex or age on EdF.

It was found that 48.57% of the study sample had delayed language development (DLD), and this may be due to the overlap in neural processes between language skills and EF. This goes with a study by Slot et al. (2018) [18], which reported results supportive of bidirectional associations between language skills and EFs in German preschool children.

More specifically, the research done by Pereira et al. (2020) [19] demonstrated a significant positive correlation between syntactic comprehension and working memory (*p*-value of < 0.001) and a weak positive correlation between syntactic comprehension and inhibitory control (*p*-value of < 0.05).

As regard the type of school, it was found that the majority of cases (71.43%) went to governmental schools as most of the children referred to our facility were enlisted in these types of schools, and this is consistent with the study by Barbosa et al. (2019) [1], which reported that 62.5% of their sample went to governmental schools.

It was also found that 82.86% of the study sample had normal mainstream education (as opposed to integrated education) as the diagnosis of SLD and EdF was not yet confirmed. The literature reviews did not present relevant information about the relation between the type of school or education and EdF.

As regards the general intelligence quotient (GIQ), it was found that the majority of the study sample (57.14%) had an average GIQ as most dyslexic children often have an average IQ; furthermore, the Stanford-Binet psychometric test evaluates the more basic cognitive processes which seem to be intact in these children. This is consistent with the findings of Locascio et al. (2010), which presented a sample that have a full-scale IQ that falls mostly in the average area (a mean of 92.07 and a standard deviation of 10.74). Furthermore, no correlation was found between the degree of EdF and the GIQ [20].

As regards ADHD, it was noticed that 71.43% of the study sample was diagnosed with ADHD with a significant positive correlation between the severity of EdF and ADHD (*p*-value of < 0.001) which may be attributed to the similarity in the neural pathways and neurotransmitter defects between ADHD and EdF, and these results are consistent with the research done by Bathelt et al. (2018) [21], which reported a significant positive correlation between ADHD and EdF severity with a *p*-value of < 0.001, and are also consistent with the results of the research done by Barkley et al. (2014) [22], which demonstrated a significant positive correlation between EdF across all its domains and ADHD.

Although all the study samples were diagnosed with both dyslexia and EdF, no correlation was found between the severity of dyslexia and EdF (*p*-value of 0.721) which may be due to the difference in structural connectivity between dyslexia and EdF; furthermore, a larger number of cases may be needed to study the correlation between these variables, and this coincides with the study done by Bathelt et al. (2018) [21], which reported a not significant correlation with a *p*-value of 0.144. On the contrary, Pascual et al. (2019) [23] reported a significant correlation between the degree of academic performance and EF with a *p*-value of < 0.05.

There was a statistically significant improvement in ADAT scores after applying the rehabilitation program with a *p*-value of < 0.001 as some components of the rehabilitation program directly address certain deficits in dyslexia such as reading comprehension and written expression. This is consistent with the findings of McClelland et al. (2018) [16], which demonstrated that the application of rehabilitation programs for building EF skills is, directly and indirectly, related to enhancing academic outcomes, and it also demonstrated that programs that focus on a specific domain (for example, EF skills) can yield improvement in other domains such as reading and math abilities.

There was a statistically significant improvement in BDEFS-CA scores across all the scale domains (SMT, SO, SR, SM, and SRE), as well as the summary score (EFSS) after applying the rehabilitation program with a *p*-value of < 0.001, and this goes with the study by Entwistle et al. (2014) [24], which reported a statistically significant improvement after the application of a training program that specifically targeted the working memory (WM) component of EFs with a *p*-value of < 0.05.

It was found that a strong positive correlation exists between the degree of improvement of dyslexia and all EF domains (SMT, SO, SR, and SRE with a *p*-value of 0.001, 0.002, 0.019, and 0.007, respectively) except for the SM domain; furthermore, the degree of improvement of the EF overall score was significantly correlated with the degree of improvement of dyslexia with a *p*-value of < 0.001 which goes with the study by Sung et al. (2018) [25], which reported a significant positive correlation between the rate of improvement of EdF and dyslexia with a *p*-value of < 0.001.

So, the rehabilitation program was effective mainly in improving EF deficits, as well as helping in the improvement of dyslexia for better academic performance in Egyptian elementary students. Limitation to this study included dropping out of some cases before completing the therapy and also poor compliance. In addition, delayed rehabilitation during the COVID-19 pandemic caused some difficulties in the proper administration of the rehabilitation program. Finally, some parents were not cooperative in applying the carryover activities in the home setting.

## Conclusion

This study proved that designing an Arabic rehabilitation program specific for EdF was effective for improving both EF deficits and dyslexia, but there is a need for further studies comparing this program to other methods of traditional interventions.

## Limitations of the study

It is recommended in further work to measure the improvement of scholastic achievement and learning process in the classroom through involvement of class teachers in the evaluation pre and post rehabilitation.

## Data Availability

The datasets used and/or analyzed during the current study are available from the corresponding author on reasonable request.

## Abbreviations

EF: Executive functions
SLD: Specific learning disorder
EdF: Executive function difficulties
CAAS: Children’s attention and adjustment survey
ADHD: Attention-deficit/hyperactivity disorder
BDEFS-CA: Barkley Deficits in Executive Functioning scale, Children and Adolescents long form
DLD: Delayed language development
